# Evaluation of the Effect of Refractive Window Drying Using Ultrasound as Pretreatment on the Preservation of the Chemical, Physical and Techno-Functional Properties of the Leaf of *Bauhinia forficata*

**DOI:** 10.3390/molecules31122058

**Published:** 2026-06-12

**Authors:** Cecilia E. Martínez-Sánchez, Ivet Gallegos-Marín, Roselis Carmona-García, Jesús Rodríguez-Miranda, Juan G. Torruco-Uco, Emmanuel de J. Ramírez-Rivera, Adriana Moreno-Rodríguez, Carolina Calderón-Chiu, Erasmo Herman-Lara

**Affiliations:** 1 Departamento de Ingeniería Química y Bioquímica, Tecnológico Nacional de México Campus Tuxtepec, Avenida Dr. Víctor Bravo Ahuja No. 561, Col. El Paraíso, Tuxtepec C.P. 68350, Oaxaca, Mexico; cecilia.ms@tuxtepec.tecnm.mx (C.E.M.-S.); roselis.cg@tuxtepec.tecnm.mx (R.C.-G.); jesus.rm@tuxtepec.tecnm.mx (J.R.-M.); juan.tu@tuxtepec.tecnm.mx (J.G.T.-U.); 2 Departamento de Ingeniería Química y Bioquímica, SECIHTI, Tecnológico Nacional de México Campus Tuxtepec, Avenida Dr. Víctor Bravo Ahuja No. 561, Col. El Paraíso, Tuxtepec C.P. 68350, Oaxaca, Mexico; ivet.gallegos@secihti.mx; 3 Departamento de Innovación Agroalimentaria Sustentable, Tecnológico Nacional de México Campus Superior de Zongolica, Km 4 Carretera Tepetitlanapa, Zongolica C.P. 95005, Veracruz, Mexico; ejramirezrivera@zongolica.tecnm.mx; 4 Facultad de Ingeniería Química, Universidad Autónoma Benito Juárez, Av. Universidad S/N Ex-Hacienda de 5 Señores, Oaxaca de Juárez C.P. 68120, Oaxaca, Mexico; amoreno.cat@uabjo.mx; 5 Departamento de Ingeniería en Industrias Alimentarias, Tecnológico Nacional de México Campus Superior de Teziutlán, Teziutlán C.P. 73960, Puebla, Mexico; carolina.cc@teziutlan.tecnm.mx

**Keywords:** *Bauhinia forficata*, bioactive compounds, refractance window drying, ultrasound, antioxidant activity, hypoglycemic activity

## Abstract

*Bauhinia forficata* leaves were subjected to ultrasonic pretreatment and subsequently dried using a refractance window (RW) and tray drying (TD). The physical, chemical, and biological properties of the dried leaves were evaluated under both drying methods, with and without ultrasound. RW combined with ultrasound (RW-US) resulted in the shortest drying time (90 min) and the lowest values of water activity (0.21), color difference (Δ*E* = 0.61), and maximum shear force (14.72 N), indicating improved drying efficiency and texture preservation. In addition, the RW-US samples exhibited the highest water solubility capacity (13.75%), water absorption capacity (5.56 g water/g dry matter), and swelling power (9.95%). With respect to structural changes, thickness showed the greatest percentage reduction during drying. The RW-US treatment also preserved bioactive compounds more effectively, yielding the highest total polyphenol content (61.96 mg GAE/g extract), flavonoid content (308.44 mg QE/g extract), antioxidant activity (60.50% by DPPH• and 70.15% by ABTS•+), and chlorophyll content (2.65 mg/g), the values of which were closest to those of fresh leaves. None of the extracts showed cytotoxic effects, with respect to hypoglycemic activity, the best treatments were RW, RW-US, and TD, which resulted in glucose reductions of 51.64%, 41.95% and 39.70%, respectively. Overall, RW-US drying preserved most of the physical, chemical, and biological properties, resulting in the production of a potential functional ingredient for foods.

## 1. Introduction

Drying is a key preservation technique used to extend shelf life and stabilize plant-derived materials; however, conventional convective methods often require long processing times, leading to the degradation of thermolabile compounds, loss of bioactive molecules, and deterioration of functional properties [1]. These limitations have driven the development of alternative drying technologies aimed at improving efficiency while preserving chemical integrity.

Refractance window (RW) drying has emerged as a promising technique due to its rapid heat transfer and relatively low operating temperatures (<98 °C). Although in this process thermal energy is transferred by conduction and convection its usefulness is minimal, since the energy transfer is mainly by infrared radiation through a thin film known as Mylar in contact with hot water, which allows efficient removal of moisture from the wet solid while minimizing thermal damage [2,3,4]. Furthermore, some additional advantages include high product quality, as it better preserves the nutritional value, bioactive compounds, vitamins, aroma, and original color of fruits, vegetables, and other foods, surpassing conventional methods such as spray drying or hot air drying. Another advantage is energy efficiency and cost-effectiveness, as it consumes less energy than freeze-drying and has lower maintenance and operating costs. It also employs short drying times, making the process fast and converting purees or liquids into powder in a matter of minutes, thus increasing productivity. Additionally, it uses moderate (low) temperatures; by not subjecting the product to high temperatures, thermal damage is avoided, which is ideal for heat-sensitive foods. Finally, its versatility and safety make it easy to dry viscous products and purees, with very low risks of contamination thanks to the plastic barrier. However, some disadvantages of refractive window drying include: limitations on product shape, higher initial costs and difficulty with sticky foods [5,6].

As a result, RW drying has been associated with improved retention of phytochemicals, including polyphenols and pigments, as well as preservation of techno-functional properties such as water absorption and solubility [5,6,7].

Ultrasound (US) has become one of the most important techniques in green chemistry and emerging technologies. Many research investigations documented the usefulness of US in a wide range of applications in food science, nanotechnology, and complementary medicine, where effective extraction of natural products is important. The application of US enhances the rates of chemical processes such as hydrolysis of herbal fibers, which reduces the time and energy consumed without affecting the quality of the final products. Overall, the use of US in herbal science has great potential to create novel chemical constructions and to be used as an innovative diagnostic system in various biomedical, food, and analytical applications [8].

To further enhance mass transfer and structural modification, ultrasound has been proposed as a pretreatment. Acoustic cavitation induces microstructural changes in plant tissues, generating microchannels that facilitate moisture diffusion and improve the release and preservation of intracellular compounds [8,9,10,11]. Therefore, this combined approach can not only contribute to better stabilization of bioactive compounds and other physical properties such as color retention, but also, in some cases, release of bioactive molecules during drying [10,11].

*Bauhinia forficata*, commonly known as “cow foot” or “cow hoof,” is one of the most extensively used Bauhinia species in traditional medicine for the management of diabetes and is often referred to as “plant insulin” [12,13]. Its hypoglycemic activity has been attributed primarily to the flavonoid kaempferitrin, which has been reported to be predominantly localized in the leaves of *B. forficata* and may serve as a chemotaxonomic marker. The potential anticancer properties of this species have also been investigated; however, studies addressing its cytotoxic activity remain limited [13,14,15]. Other reported therapeutic applications of *Bauhinia forficata* include the management of cardiovascular diseases and renal disorders [15]. In addition, its biological potential has been extensively documented, particularly regarding its antioxidant [15,16] and anti-inflammatory activities [16,17,18]. Although refractance window (RW) drying has been extensively applied to fruits, vegetables, and protein-based systems and its combination with emerging technologies has been explored, its application to medicinal plants remains largely unreported. In particular, no studies have systematically investigated the impact of ultrasound as pretreatment in RW drying on the preservation of bioactive compounds and their associated biological activities in medicinal plant matrices. Therefore, for the first time, this study aimed to evaluate, the effects of ultrasound-assisted RW drying on the physicochemical properties and, critically, on the biological activities—specifically, cytotoxicity and hypoglycemic activity—of *Bauhinia forficata* leaves, providing new insights into the stabilization of bioactive molecules during drying.

## 2. Results and Discussion

### 2.1. Drying Kinetics

The initial weight of the fresh samples per batch had an average of 80.02 g corresponding to an initial moisture content of these samples of 0.9696 g water/g dry solids.

**Figure 1:**
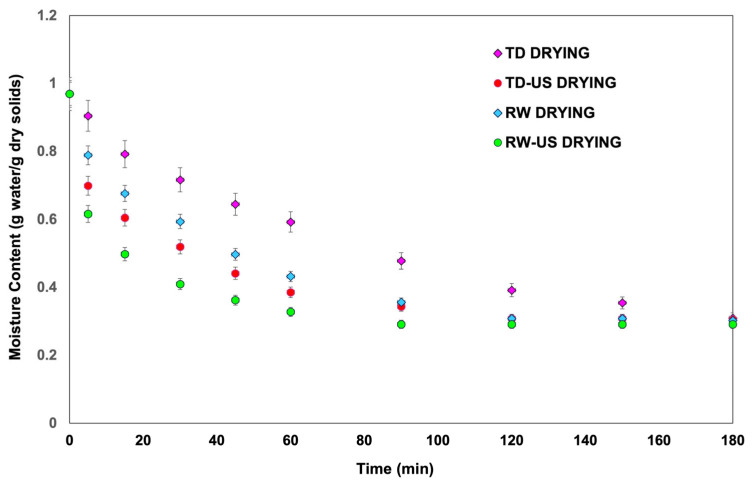
Drying kinetics at 70 °C of *B. forficata* leaves under different drying treatments. RW = Refractive Window Drying, RW-US = Refractive Window-Ultrasound Drying, TD = Tray Drying, and TD-US = Tray-Ultrasound Drying.

The drying kinetics are presented in Figure 1. It was observed that at the same drying time, specifically at 90 min, the moisture contents were 0.4781, 0.3565, 0.3436 and 0.2915 g water/g dry solids which correspond to the drying treatments of TD, RW, TD-US and RW-US respectively. The lowest moisture contents were obtained in the drying treatments in which US was used as a pretreatment and RW compared to TD. However, of these drying treatments, RW-US was the only one that reached equilibrium moisture content (EMC) of 0.2915 g water/g dry solids during this time as shown in Table 1. Establishing EMC as the minimum amount of water retained by the leaves of *B. forficata* at a constant temperature for a certain time. In this study, Figure 1 shows when the drying kinetics curve becomes asymptotic or in a direction parallel to the time axis. On the other hand, we can also establish that at 90 min, the drying kinetics with the TD-US and RW drying treatments, although they present different moisture content values, did not show significant differences (*p* > 0.05). The TD-US and RW drying treatments reached their EMC at 120 min with no significant difference with RW-US with values of 0.3084 and 0.3086 g water/g dry solids respectively. Finally, the TD reached its EMC of 0.3098 g water/g dry solids at 180 min with a value without significant differences compared to the other drying treatments used but at different drying times. These values and their significance are presented in Table 1. The quality parameters were evaluated in each drying sample at the times when the EMC were reached, in order to establish the damage or retention of these parameters by the individual drying times in each treatment in the leaves of *Bauhinia forficata*.

The shorter drying times observed in the ultrasound-treated samples could be attributed to greater moisture diffusion, probably as a result of microstructural modifications such as pore formation induced by acoustic cavitation as reported by Wang et al. [11]. In general, the variation in moisture content over time reflects distinct drying behaviors between ultrasound-treated and untreated *B. forficata* leaves.

The drying kinetics revealed that the reduction in moisture content was fastest during the 40–60 min drying period, specifically in the RW, RW-US, and TD-US treatments, but not in TD. During the 60–120 min drying period, the first three treatments reached their EMC more slowly, with RW-US being the first to reach this value, as previously mentioned. This was invariably due to the ultrasound treatment, since when only RW was used to dry the *B. forficata leaves*, the EMC was not reached until after 90 min, nor was it reached in the TD-US treatment. However, it is also possible to establish that the ultrasound treatment in TD significantly reduced the drying time, as TD without ultrasound was the slowest drying method, taking 3 h to reach its EMC. This, along with the type of heat transfer used, resulted in a serious deterioration of the quality parameters of the material used in this study.

Emelike & Akusu [19] reported similar behavior to this study for leafy materials such as *Ocimum gratissimum*, *Vernonia amygdalina*, *Moringa oleifera*, and *Heinsia crinita*. In this context, the drying performance was strongly influenced by the initial moisture content and the internal structure of the plant matrix, which control the effective moisture diffusivity.

Carcel et al. [20] reported distinct drying kinetics in red pepper under different temperature and ultrasound conditions, demonstrating that mass transfer is highly dependent on both process parameters and material characteristics. Furthermore, Yilmaz et al. [21] reported a significant reduction in drying time for ultrasound-treated carrot slices, attributed to enhanced effective diffusivity resulting from microstructural changes induced by acoustic cavitation. These findings support the role of ultrasound in facilitating moisture transport through the formation of microchannels, thereby accelerating the drying process as established in this scientific research and as reported by Gouda et al. [8] for other types of vegetable materials.

Wang et al. [11] reported that the ultrasound strengthened air impingement drying improved drying efficiency and physicochemical quality of *Chaenomeles sinensis* slices. These findings suggested that appropriate ultrasonic pretreatment facilitated the release and retention of bioactive compounds by altering the microstructure and crystallinity, as well as shortening drying duration. In summary, ultrasound-assisted air impingement drying is an effective approach to processing *C. sinensis* slices with the improvement of drying efficiency and physicochemical properties of products.

### 2.2. Physical Properties

#### 2.2.1. Color Parameters of *Bauhinia forficata* Leaves

The determination of color parameters was performed on samples dried by RW, RW-US, TD, TD-US, and on fresh samples.

Table 1 presents the color parameters for L*, a*, and b* of *B. forficata* leaves under the different drying treatments. Fresh samples exhibited values of L* = 12.77, a* = 4.26, and b* = 35.13; the RW-US treatment showed L*, a*, and b* values of 13.04, 3.93, and 35.57; and the RW showed L*, a*, and b* values of 12.90, 4.67, and 34.84, respectively. No significant differences were detected between the RW-US and RW treatments or relative to the fresh samples (*p* > 0.05), indicating effective preservation of color attributes.

This behavior suggests limited degradation of chlorophyll and associated pigments, likely due to the mild thermal conditions and short drying times characteristic of RW drying. The predominance of infrared heat transfer, coupled with rapid moisture removal, may reduce pigment oxidation and enzymatic browning, thereby minimizing overall color changes (Δ*E*). In contrast, compared with the fresh leaves, the TD-US samples presented L*, a*, and b* values of 11.39, 5.72, and 35.14, respectively, whereas the TD samples exhibited more pronounced deviations of 8.00, 9.94, and 30.05, respectively (*p* < 0.05). The lower L* values and increased a* values suggest darkening and a shift toward reddish tones, which are likely associated with chlorophyll degradation and the formation of pheophytins under prolonged thermal exposure. Wang et al. [11] stated that the ultrasonic time and powers had significant impacts on the color parameters of dried *C. sinensis* slices. All ultrasound-pretreated samples showed slightly lower L* values (71.28–73.79) than the control, although still higher than those of fresh tissues. It might be attributed to the ability of ultrasound pretreatment to partially preserve the original color by mitigating excessive brightness, which was typically caused by structural collapse during drying. However, these values are completely different from those obtained in this study, perhaps due to the dimensions of the plant leaf and the different operating conditions of the ultrasound.

Overall, the RW-based treatments better preserved the color quality of *B. forficata* leaves, maintaining color parameters closer to those of the fresh material.

Gouda et al. [8] established regarding the L* parameter, that convection drying treatments exhibited a marked decrease in luminosity, indicative of sample darkening. This behavior is primarily associated with chlorophyll degradation through pheophytinization, a process in which the central Mg^2+^ ion of the chlorophyll molecule is replaced by H^+^ under thermal and acidic conditions. This transformation leads to the formation of pheophytins and, at higher temperatures or prolonged exposure, pyropheophytins. These derivatives are characterized by dull olive-brown tones, resulting in a loss of the characteristic bright green color and a concomitant increase in a* values, reflecting a shift toward reddish hues in leaves of vegetable materials.

Additionally, non-enzymatic browning reactions, such as Maillard reactions and pigment oxidation, may further contribute to color deterioration under extended drying times how was reported by Wang et al. [11]. This was not the case in this study, where the US associated with subsequent RW drying or RW not US maintained the color parameters.

In terms of the b* parameter, the TD-RW, RW and TD-US treatments resulted in b* values comparable to those of the fresh sample, with no statistically significant differences (*p* > 0.05). This preservation of the green color of the leaves can be attributed to the shorter drying times with infrared radiation for both the RW and RW-US treatments, associated with pretreatment with ultrasound, which limits the degradation of the pigment and reduces the magnitude of the thermal and oxidative reactions that affect carotenoids and related compounds. In the tray drying treatments (TD and TD-US), heat transfer occurred primarily by convection, which explains the more pronounced alterations in leaf pigments compared with those in the RW and RW-US treatments, where color parameters remained closer to those of fresh leaves. The extended exposure to hot air during convective drying promotes chlorophyll degradation and pigment oxidation, leading to greater color deviations.

Wang et al. [11] reported the highest a* value (4.44) was observed at 200 W, whereas the moderate treatment (40 min and 160 W) yielded a substantially lower a* value of 2.91, reflecting better color retention. These differences could be attributed to variations in pigment exposure and oxidation during dehydration. The b* values varied significantly across different treatments. The control group exhibited the highest b* value of 23.80, indicating that excessive drying time might lead to severe color deterioration *Chaenomeles sinensis*.

Ali et al. [22] reported L*, a*, and b* values of 40.78, −4.20, and 6.33 for fresh *Moringa oleifera* leaves, which changed to 36.52, −3.14, and 11.95, respectively after oven drying at 60 °C. These results indicate a decrease in luminosity and an increase in b* (yellowing), attributed to convective heat transfer and pigment degradation. The results found in this type of *M. oleifera* leaves are completely different from those reported in this research for *B. forficata*, perhaps due to the different operating conditions of both the US as pretreatment and the RW drying.

This study demonstrated that color changes were less than one unit or low in the RW-US and RW treatments, with Δ*E* no significant differences between these treatments (*p* > 0.05). This suggests that these treatments did not significantly alter the color of fresh *Bauhinia forficata* leaves. However, the TD-US and TD treatments did show values of 2.01 and 8.99, respectively, with significant differences (*p* < 0.05) between these treatments, as well as significant differences compared to the first two treatments using RW-US and RW drying.

Wang et al. [11] found that the drying effect of changes in color parameters of L*, a* and b* in *Chaenomeles sinensis* slices were different to those of this study. This behavior was reflected in the total difference in color change (Δ*E*), indicating that perceivable to pronounced visual changes occurred. In contrast, RW-based treatments resulted in lower Δ*E* values, suggesting minimal color alteration and improved color quality retention.

On the other hand, Wang et al. [23] observed significantly higher Δ*E* values in *Zanthoxylum bungeanum* leaves subjected to conventional drying methods than in those obtained in the present study, particularly under RW and ultrasound-assisted conditions, which demonstrated superior color preservation.

#### 2.2.2. Maximum Shear Force

**Figure 2:**
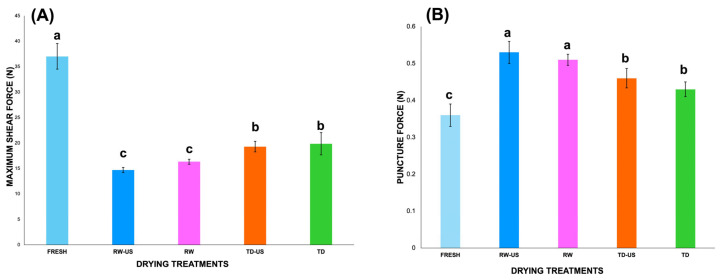
Effect of drying treatments on maximum shear force (MSF) (**A**) and puncture force (PF) (**B**) of *Bauhinia forficata* leaves. Values are mean ± SD (n = 4). Different lowercase letters indicate significant differences (LSD, *p* < 0.05).

Dehydration significantly alters the mechanical properties of plant tissues as a result of moisture removal and associated structural changes The maximum shear force (MSF) values for each treatment are shown in Figure 2A. Fresh leaves presented the highest MSF (36.04 N), followed by RW-US (14.72 N), RW (16.35 N), TD-US (19.31 N), and TD (19.87 N).

No significant differences were detected between RW-US and RW (*p* > 0.05) or between TD-US and TD (*p* > 0.05). The higher MSF of fresh samples is attributed to the presence of turgor pressure and intact cellular structure, where water acts as a plasticizing agent, maintaining cell wall rigidity and resistance to deformation. Upon drying, the loss of intracellular water reduces the turgor pressure and promotes structural collapse, leading to decreased mechanical strength.

The lowest MSF values were observed in the RW-US and RW samples, suggesting greater alteration of the cellular structure, increasing porosity and weakening the cellular matrix in *B. forficata* leaves, thus reducing resistance to shear forces. This behavior can be explained primarily by the effect of infrared radiation on the reduction in water content due to RW drying, since the RW treatment with ultrasound, despite showing a lower numerical value as previously described, did not represent a significant difference between the two treatments.

Treatments based on TD with or without ultrasound also showed no significant differences between them. However, higher MSF results were obtained than those obtained by the two RW treatments with significant differences between these two types of drying, probably due to longer drying times and the form of convective energy transfer where hot air is used, which as was already emphasized, is a more aggressive type of drying that produces a slow diffusion of water to the surface of *the B. forficata* leaves causing a compaction of the cellular tissue, less development of porosity and greater resistance to shear force.

It can be established that the reduction in MSF was due to the different type of energy transfer employed in both types of drying by RW and TD and not due to the effect of ultrasound in both cases.

From a techno-functional perspective, lower MSF values are associated with softer textures, which may be advantageous for applications requiring rapid rehydration or improved palatability. Conversely, higher MSF values indicate firmer structures, which may be desirable for maintaining structural integrity during handling and storage.

These results, although from a different raw material than this study, show a similar behavior with Zhao et al. [24], who reported MSF values between 14 and 18 N for oat kernel, highlighting the strong dependence of mechanical properties on moisture content and internal structure.

Hadidi et al. [25] showed that ultrasound-assisted extraction improved the yield and bioaccessibility of saponins from alfalfa leaves by enhancing mass transfer and cell wall disruption through acoustic cavitation. Similarly, the present study demonstrated that ultrasonic pretreatment combined with refractance window drying (RW-US) improved drying efficiency and preserved bioactive compounds, antioxidant activity, and functional properties in *Bauhinia forficata* leaves. Although focused on different processes, both studies confirm that ultrasound is an effective green technology for improving the processing and quality of plant-based materials, supporting its application in functional foods and nutraceuticals.

Zhao et al. [26] also showed that ultrasound-assisted enzymatic extraction significantly improved the extraction of polysaccharides and bioactive compounds from *Turpiniae folium*. The optimized conditions increased extraction efficiency, demonstrating that ultrasound enhanced cell wall disruption and mass transfer, resulting in higher polysaccharide yield. Similarly, in the present work, ultrasound pretreatment combined with refractance window drying (RW-US) improved the drying performance and preservation of *Bauhinia forficata* leaves. RW-US reduced drying time and better-preserved bioactive compounds, antioxidant activity, chlorophyll content, and functional properties compared to the other treatments.

#### 2.2.3. Puncture Force

Fresh leaves presented the lowest value (0.35 N) in puncture force (PF) testing, whereas dried samples presented higher resistance, with values of 0.53, 0.51, 0.46, and 0.43 N for RW-US, RW, TD-US, and TD treatments, respectively. The increase in PF after drying reflects greater resistance to localized deformation, which can be attributed to moisture loss and subsequent structural rearrangement of the leaf matrix.

From a mechanistic perspective, dehydration reduces cellular turgor pressure and promotes cell wall consolidation, leading to increased rigidity at the point of penetration. Additionally, structural shrinkage and densification of cell wall polymers (e.g., cellulose and hemicellulose) contribute to increased puncture resistance. In the RW-US and RW treatments, the slightly higher PF values may also be influenced by the formation of a more compact surface layer due to rapid moisture removal.

Compared with the MSF results, an inverse trend is observed: when the PF increases after drying, the MSF decreases. This suggests that drying induces heterogeneous structural modifications, where the surface becomes more resistant to localized penetration, whereas the internal structure becomes more porous and mechanically weakened. Such behavior is consistent with the formation of microchannels and increased porosity, particularly in ultrasound-assisted treatments.

These combined effects highlight the complex relationships among the drying conditions, microstructure, and texture, which ultimately influence the functional performance of the dried material.

Compared with the MSF, the puncture force (PF) exhibited an inverse trend, as the fresh sample had the lowest PF, whereas all the dried samples treatments required higher forces, indicating increased resistance to localized penetration (Figure 2B). This behavior is associated with structural changes induced by dehydration, particularly surface densification and reduced moisture content, which increase resistance at the point of contact.

The RW-based treatments (RW-US and RW presented the highest PF values, although no significant differences were observed between them (*p* > 0.05). In contrast, the TD-US and TD treatments resulted in lower PF values, with no significant differences between these treatments (*p* > 0.05). The higher PF observed in the RW treatments may be attributed to rapid surface dehydration, leading to the formation of a more compact outer layer.

These results, despite using a different raw material or food than that of this scientific investigation, show similar behavior to those reported by Alrimawi et al. [7], who observed PF values in the range of 0.24–0.28 N for rice starch films.

Hernandez-Santos et al. [27] reported higher shear force values in relation to this research employing dried carrot slices of 2 mm thickness processed by RW, which supports the influence of the RW drying method on mechanical strength.

### 2.3. Techno-Functional Properties of Bauhinia forficata Leaf Powder

#### 2.3.1. Water Absorption Capacity and Water Solubility Capacity

Although both concepts involve water and materials, they refer to different physical processes: solubility implies complete dissolution (forming a homogeneous mixture), while absorption implies physical retention (water enters the material’s structure without disappearing).

The rehydration behavior of dried *B. forficata* leaves is closely linked to the drying kinetics and the resulting microstructural modifications. Faster moisture removal, as observed in the RW-US treatments, promoted the formation of a more porous structure due to rapid vapor flux and internal pressure gradients. These structural changes facilitate mass transfer during drying and subsequently influence water absorption during rehydration as observed by Alrimawi et al. [7], Zhao et al. [24] and Hernández-Santos et al. [27] in rice starch films, oat kernel and carrot slices respectively.

As shown in Table 2, the water absorption capacity (WAC) ranged from 5.36 to 5.56 g water/g dry matter, with no significant differences among the treatments (*p* > 0.05). Despite the differences in drying rates, this result indicates that the overall capillary network and hydrophilic binding sites were largely preserved. From a structural standpoint, WAC depends on pore connectivity and the ability of the matrix to retain water via capillary forces and hydrogen bonding. The relatively stable WAC values suggest that although ultrasound and RW enhanced moisture diffusion, they did not induce excessive collapse or degradation of the matrix.

These findings are consistent with the texture results. The lower MSF observed in the RW-US samples indicates increased porosity and reduced mechanical resistance, which typically favor water penetration. However, the simultaneous increase in puncture force (PF) suggests surface densification, which is likely caused by rapid drying of the outer layers. This dual behavior—a porous internal structure with a more compact surface—explains why WAC remained stable while the other properties were modified.

In contrast, the water solubility capacity (WSC) was more sensitive to processing conditions and directly related to structural disruption. The highest values were obtained in the ultrasound-assisted treatments (13.75% for RW-US and 12.11% for TD-US), reflecting the enhanced release of soluble compounds due to cavitation-induced cell wall rupture. This trend aligns with the observed decrease in MSF, confirming that ultrasound promotes matrix weakening and increases the availability of low-molecular-weight fractions.

Byarugaba et al. [28] demonstrated that RW drying is an effective technology for preserving the quality and safety of *Amaranthus cruentus* leaves. The combination of fermentation and RW drying produced shelf-stable products with improved sensory acceptability. These findings highlight the potential of RW drying for maintaining nutritional and functional properties in African indigenous vegetables. Similarly, the present study showed that RW drying, especially when combined with ultrasonic pretreatment (RW-US), preserved the functional properties of *Bauhinia forficata* leaves specifically in WSC.

Overall, the interplay between drying kinetics and structural modification determines the techno-functional performance of the dried material. Rapid drying (RW-US) enhances internal porosity and solute accessibility while maintaining sufficient structural integrity for water retention. This balance is desirable for applications requiring rapid rehydration, improved dispersibility, and high functional value. Nobossé et al. [29] reported comparable behavior to this study in leaf matrices such as *Moringa oleifera*, which supports the general applicability of these mechanisms, specifically in the techno-functional properties of WAC and WSC, which remained acceptable in the leaves of *B. forficata*.

#### 2.3.2. Swelling Power

Swelling power (SP) reflects the ability of *B. forficata* leaf powders to absorb water and increase in volume and is closely related to the structural integrity and availability of hydrophilic sites within the matrix. Temperature does affect the swelling power of any dry material, and at higher treatment temperatures, this parameter increases; that is, the swelling power of the dry material will be greater, as shown in Table 2 in all drying treatments. Vaishnavi et al. [30] reported this trend in edible vegetable films.

However, as shown in Table 2, temperature did not significantly affect SP in most treatments (*p* > 0.05), except for TD-US above 70 °C and TD at 90 °C, where noticeable decreases were observed. The SP values ranged from 9.02 to 9.95 (RW-US), 8.51 to 9.61 (RW), 7.80 to 9.27 (TD-US), and 6.77 to 8.85 (TD), indicating superior swelling capacity in the RW-based treatments. The shorter drying times in RW treatments with or without ultrasound may explain this fact, since infrared radiation affects the fiber characteristics of the dried leaves of *B. forficata* less. In addition, the fact that a structural change at the cellular level, gelatinization and protein denaturation could be less in relation to convective drying with hot air in TD and TD-US treatments was also observed by Aidoo et al. [31] in cassava flour and starch and Raghavi et al. [6] observed this trend in some foods when RW drying was applied.

Comparative analysis revealed significant differences between drying methods at 60 and 70 °C, even in the absence of ultrasound, suggesting that heat transfer mechanisms play a key role in determining structural functionality. At higher temperatures (80–90 °C), the SP values became more homogeneous, with significant differences observed mainly between the tray drying and the RW-based treatments, indicating a convergence of structural effects under more severe thermal conditions. From a mechanistic perspective, SP is governed by matrix porosity, polymer relaxation, and the availability of functional groups capable of forming hydrogen bonds with water. Zhao et al. [24] explained theses effects in mechanical properties for shearing breakage of oat kernel. Ultrasound pretreatment and rapid drying likely promote partial disruption of the plant structure, enhancing the accessibility of hydrophilic components such as polysaccharides and proteins. During drying, the removal of bound water exposes these functional groups, enabling renewed interactions with water upon rehydration. Gouda et al. [8] observed this phenomenon in vegetable materials.

Overall, the higher SP values observed in RW and the RW-US treatments suggest better preservation of structural features that favor water uptake and volumetric expansion, which is consistent with their drying kinetics and microstructural characteristics.

### 2.4. Shrinkage Coefficient

Shrinkage, expressed as volume reduction, is a key physical change associated with moisture removal during drying and reflects structural collapse of the plant matrix. Table 3 summarizes the dimensional changes in *B. forficata* leaves under the different treatments. The greatest reduction was observed in thickness, with values of 32.42%, 30.65%, 32.12%, and 31.28% for RW-US, RW, TD-US, and TD, respectively. In contrast, width and length decreased less, ranging from 3.35 to 3.79% and from 4.23 to 4.73%, respectively. All dimensional changes were statistically significant among the treatments (*p* < 0.05).

The pronounced reduction in thickness can be attributed to the collapse of internal cellular structures and the loss of turgor pressure during drying, which primarily affects the direction perpendicular to the leaf surface. The relatively small changes in width and length suggest that the leaf’s planar structure provides some resistance to shrinkage along these axes. However, the overall deformation leads to an irregular morphology and partial loss of the native laminar structure. Therefore, there was practically no linear deformation.

This anisotropic shrinkage behavior is consistent with previous reports. Essaghi et al. [32] observed similar results to this study in leaves of *Quercus suber*, reporting reductions of 30.27% in thickness, 6.39% in width, and 2.36% in length under natural drying conditions. These findings support the idea that thickness is the dimension most sensitive to drying-induced structural changes in leaf materials.

Rashid et al. [33] found that when slices of sweet potatoes were dried with hot air, they underwent deformations resulting in a reduction in diameter, but not in thickness. Although they are different food materials, the behavior was similar to that observed in the present study.

### 2.5. Chemical Properties

#### 2.5.1. Water Activity

Water activity (a_w_) is a key parameter governing microbial stability, as values above approximately 0.60 generally permit the growth of spoilage microorganisms. Fresh *B. forficata* leaves presented a high a_w_ (0.92), indicating high susceptibility to microbial proliferation and limited shelf life. Emelike & Akusu [19], in their review of comparative effects of drying on selected vegetables, support similar assertions to those in this scientific work.

As shown in Table 1, the units of a_w_ were dimensionless and additionally all drying treatments significantly reduced a_w_ with respect to fresh leaves, with values reaching approximately 0.21–0.34 depending on the process. Ultrasound-assisted treatments (RW-US and TD-US) consistently achieved lower a_w_ values than their non-ultrasound counterparts (RW and TD), reflecting enhanced moisture removal. This improvement can be attributed to acoustic cavitation, which promotes microchannel formation and accelerates internal mass transfer. Pandiselvam et al. [10] in their review of the individual and interactive effect of ultrasound pretreatment on drying kinetics and biochemical qualities of food, such as edible rice starch films and other fruit and vegetables slices, reported findings similar to those obtained in this study.

A comparative analysis revealed that RW drying without ultrasound resulted in a_w_ values statistically similar to those of TD-US (*p* > 0.05), considering the same final drying times, highlighting the superior efficiency of RW in terms of heat and mass transfer. The lowest a_w_ values (≈0.21) observed in RW-US indicate a synergistic effect between rapid infrared radiation heat transfer and ultrasound-induced cellular structural modification, that is, the formation of microchannels in the leaves of *B. forficata*. Hernandez-Santos et al. [27] reported similar behaviors in the relationship between high and low water activities with high and lower moisture contents respectively.

The reduction in a_w_ to values well below 0.60 indicated that all the dried samples were microbiologically stable, with extended shelf life and a reduced risk of spoilage. From an application perspective, these low a_w_ levels enable storage under ambient conditions; however, to maintain quality and prevent moisture reabsorption, appropriate packaging is needed. Materials with low water vapor permeability, such as multilayer barrier films or vacuum-sealed packaging, are recommended. Additionally, storage in low-humidity environments is essential for preserving the techno-functional properties and preventing caking or structural degradation over time. Vaishnavi et al. [30] and Rashid et al. [33] reported of these findings on edible coatings and dried sweet potatoes respectively, preserving the techno-functional properties and nutritional quality.

#### 2.5.2. Total Polyphenol and Total Flavonoid Contents

The leaves of *Bauhinia forficata* are widely recognized for their medicinal properties, largely attributed to their high abundances of polyphenols and flavonoids [11,34]. The total polyphenol content (TPC) results are presented in Table 4, which reveals significant differences among all drying treatments (*p* < 0.05). The highest TPC values were obtained in the ultrasound-assisted treatments, particularly for RW-US (61.96 mg GAE/g extract), followed by TD-US (37.34 mg GAE/g extract) and RW (33.67 mg GAE/g extract). These enhancements can be attributed to the mechanical effects of ultrasound, such as cavitation-induced cell disruption, which promotes the release of phenolic compounds previously located in soluble fractions or bound to cell wall components.

Notably, polyphenols were most effectively preserved and concentrated in the RW-US treatment, suggesting a synergistic effect between rapid drying and ultrasound-induced structural modification. In contrast, flavonoids were more widely distributed across treatments, with higher concentrations observed in RW-US, RW, and TD-US samples. This behavior may be related to the naturally high flavonoid content of *B. forficata*, as previously reported by Miceli et al. [17], who identified compounds such as catechin, rutin, isoquercetin, naringin, kaempferol, and myricetin, as well as phenolic acids including gallic and chlorogenic acids.

Byarugaba et al. [28] showed that RW drying is an effective technology for preserving the quality of *Amaranthus cruentus* leaves. The combination of fermentation and RW drying produced shelf-stable products with improved protein digestibility and mineral bioaccessibility, while non-fermented RW-dried leaves retained higher levels of β-carotene, polyphenols, flavonoids, and sensory acceptability. These findings highlight the potential of RW drying for maintaining nutritional and functional properties in African indigenous vegetables. Similarly, the present study showed that RW drying, especially when combined with ultrasonic pretreatment (RW-US), improved the preservation of physical, chemical, and biological properties of *Bauhinia forficata* leaves. RW-US reduced drying time and better-preserved bioactive compounds, antioxidant activity, chlorophyll content, and functional properties compared to tray drying.

The total flavonoid content (TFC) (Table 4) values were consistently greater than the TPC values across all the treatments. These differences can be explained by methodological and compositional factors. The Folin–Ciocalteu assay used for calculating TPC primarily detects low- to intermediate-molecular-weight phenolics extractable by solvents, whereas a significant fraction of the polyphenols in *B. forficata* may be polymerized or bound to structural components such as cellulose, hemicellulose, and lignin, limiting their extractability and reactivity. In contrast, the aluminum chloride method used for TFC calculations specifically results in the formation of stable complexes with flavonoids, enabling more efficient quantification of these compounds.

Furthermore, ultrasound pretreatment enhances cell lysis, increasing the availability of both phenolic and flavonoid compounds for extraction and detection. Comparable behavior was reported by Palsikowski et al. [35], who found TPC values of 45.69 mg GAE/g extract and TFC values of 58.51 mg QE/g extract in *B. forficata* subsp. *pruinosa*. Although the absolute values differ, a similar trend of higher flavonoid content relative to total polyphenols was observed, which may be attributed to the intrinsic chemical composition of the leaf matrix.

#### 2.5.3. Antioxidant Activity Determined by DPPH• (2,2-Diphenyl-1-picrylhydrazyl) and ABTS•+ (2,2′-Azino-bis(3-ethylbenzothiazoline-6-sulfonic acid)) Assays

**Figure 3:**
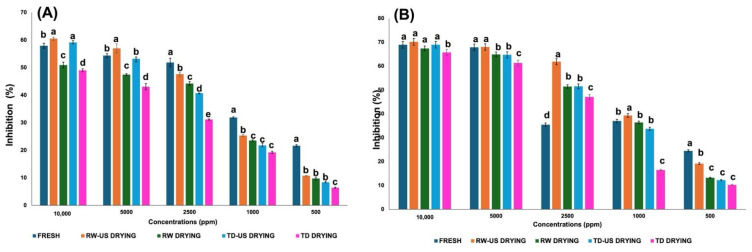
Evaluation of the different drying treatments in terms of % inhibition using the DPPH• method (**A**) and ABTS•+ method (**B**). Different letters at the same concentration indicate significant differences (LSD, *p* < 0.05).

Antioxidant activity (AA) was evaluated over a concentration range of 500–10,000 ppm. As shown in Figure 3A, the DPPH• radical scavenging activity increased with increasing extract concentration, reaching maximum inhibition at 10,000 ppm. The greatest inhibition percentage was observed for RW-US (60.50%), followed by TD-US (59.14%), fresh leaves (57.95%), RW (50.93%), and TD (49.01%). No significant differences were detected among the ultrasound-assisted treatments (*p* > 0.05), whereas significant differences were detected among the non-ultrasound treatments (*p* < 0.05). The enhanced activity levels in the in ultrasound-treated samples, which in some cases exceeded those of the fresh extracts, can be attributed to cavitation-induced cell disruption, facilitating the release of bioactive compounds available for reaction with DPPH• radicals. Adebiyi et al. [36] found comparable inhibition values (~60%) have been reported for *Grewia carpinifolia* leaves using the same method.

The ABTS•+ assay (Figure 3B) showed that the radical possessed higher antioxidant activity compared with DPPH• across all the treatments, which is consistent with its ability to react with both hydrophilic and lipophilic compounds. At 10,000 ppm, the inhibition values were 65.68% (fresh), 70.15% (RW-US), 67.38% (RW), 69.85% (TD-US), and 65.68% (TD). No significant differences were detected among the RW-US, RW, and TD-US treatments (*p* > 0.05), although the remaining treatments differed significantly (*p* < 0.05).

The results of Miceli et al. [17], as well as this study, demonstrated that antioxidant activity increased with concentration and was higher in ultrasound-assisted treatments, reflecting improved extractability of the bioactive compounds. This activity can be largely attributed to flavonoids such as kaempferol and quercetin derivatives, previously identified in *B. forficata* leaf extract. The antiradical capacities of these compounds are strongly influenced by their chemical structures, particularly the number and position of hydroxyl groups and the presence of a conjugated 2,3-double bond with a 4-oxo function in the C ring, which enhances electron delocalization [32]. In addition, flavonoids with higher levels of hydroxylation, such as quercetin and myricetin, exhibit greater radical-scavenging capacity against both DPPH• and ABTS•+ radicals [33].

It is also important to consider that antioxidant activity arises from synergistic interactions among multiple phytochemicals, including polyphenols and flavonoids, rather than from the action of individual compounds alone.

### 2.6. Chlorophyll Content

Chlorophyll, a key pigment responsible for the green coloration of plants, is highly sensitive to processing conditions [37]. As shown in Table 4, drying significantly affected the chlorophyll content (*p* < 0.05), with values of 1.59, 2.65, 1.88, 2.03, and 1.72 mg/g for the fresh, RW-US, RW, TD-US, and TD treatments, respectively. Compared with fresh leaves, all the dried samples presented higher chlorophyll concentrations, which can be attributed to the removal of water and the consequent concentration of solid components.

Among the treatments, RW-US resulted in the highest chlorophyll content, followed by TD-US, indicating that ultrasound pretreatment enhances pigment retention and/or extractability. This effect is likely associated with cavitation-induced disruption of cellular structures, including chloroplast membranes, which facilitates the release of chlorophyll molecules bound to protein complexes and membrane systems via the phytol chain. Additionally, ultrasound may partially modify structural components such as cellulose, hemicellulose, lignin, and associated proteins, increasing pigment accessibility during extraction.

The observed trends are consistent with the chlorophyll retention values (Table 4), reflecting the combined effects of concentrations and preservation during drying. RW drying, which relies on infrared radiation, enables rapid and uniform heat transfer, minimizing thermal degradation and preserving pigment integrity. In contrast, tray drying involves convective heat transfer, which may lead to greater pigment degradation; however, the application of ultrasound pretreatment mitigates this effect by enhancing mass transfer and reducing drying time.

Similar behavior has been reported by Emelike and Akusu [19], who reported increased chlorophyll concentration in dried *Moringa oleifera* leaves (1.13–1.73 mg/g), along with a retention of approximately 35%. These findings highlight the importance of preserving chlorophyll content, as it is directly associated with product quality and consumer acceptance in leafy food products. Furthermore, chlorophyll retention is influenced by both processing conditions and the intrinsic characteristics of the plant matrix.

Comparable behavior to this scientific research was informed by Byarugaba et al. [28] that demonstrated that RW drying is an effective technology for preserving the chemical quality of *Amaranthus cruentus* leaves specifically in chlorophyll content. Although the studies used different pretreatments and plant materials, both works confirm that RW drying is a promising green technology for processing leafy materials while preserving nutritional and bioactive compounds. Furthermore, both studies demonstrate that combining RW drying with complementary technologies, such as fermentation or ultrasound, can further enhance product quality, functionality, and potential industrial applications in food and nutraceutical products.

### 2.7. Biological Activity

#### 2.7.1. Cytotoxicity Assay

Plant-derived extracts may offer therapeutic benefits; however, their safety must be ensured by confirming the absence of cytotoxic effects on normal cellular functions [35]. The cytotoxicity of *B. forficata* leaf extracts was evaluated using the J774 murine macrophage line, and the results are presented in Table 5. All the samples, including the fresh and dried samples, presented CC_50_ values > 200 µg/mL, indicating that no cytotoxic effects occurred, as values above 100 µg/mL are generally considered noncytotoxic.

These findings demonstrate that none of the evaluated treatments compromised cell viability, suggesting that the drying processes and ultrasound pretreatment did not generate harmful compounds. The absence of cytotoxicity may also be associated with the presence of bioactive compounds, such as polyphenols and flavonoids, which can exert protective or modulatory effects on cellular metabolism. In some cases, these compounds may stimulate cellular activity or modulate the expression of inflammatory mediators, including cytokines and chemokines, which play key roles in immune responses [36]. However, importantly, excessive or dysregulated inflammatory responses may lead to adverse effects.

The noncytotoxic nature of the extracts suggests that *B. forficata* leaves are safe for potential use as functional food ingredients. Similar results have been reported for other plant extracts. For instance, Khaliq et al. [38] reported CC_50_ values > 100 µg/mL for *Salvadora oleoides* extracts in J774 cells, indicating low cytotoxicity. In contrast, Silva et al. [39] reported cytotoxic effects of *B. forficata* lectins on MCF-7 breast cancer cells, with CC_50_ values < 100 µg/mL. The differences observed among studies may be attributed to variations in extract composition, extraction methods, and the type of cell line used.

Mohamed [40] showed the regulation of the macrophage cellular response by *Clinacanthus nutans* leaves extracts in J774.2 macrophages. All extracts did not show any significant cytotoxic effect towards J774.2 macrophages within the extract concentration range tested (cell viability > 50%). These results showed lower cytotoxicity values than those studied in this research with *B. forficata*.

Eapen et al. [41] demonstrated that RW drying effectively preserved the phytochemical and biological properties of *Centella asiatica* leaves, particularly at 90 °C. The RW-dried samples showed high antioxidant activity, phenolic and flavonoid contents, chlorophyll content and cytotoxicity assay. Although the studies evaluated different plant materials, both works confirm that RW drying is an efficient technology for preserving phytochemicals and biological activity in medicinal leaves. Furthermore, the present work suggests that combining RW drying with ultrasound pretreatment can further improve drying efficiency and quality retention, reinforcing the potential of these technologies for developing functional foods and nutraceutical products.

Overall, these results support the safety profile of *B. forficata* leaf extracts and reinforce their potential application in food and nutraceutical formulations.

#### 2.7.2. Hypoglycemic Activity

Within the *Bauhinia* genus, *B. forficata* is widely recognized for its antidiabetic properties and is commonly referred to as “plant insulin” in traditional Brazilian traditional medicine [18]. The hypoglycemic activity results obtained in this study are presented in Table 5. Among the evaluated treatments, RW exhibited the highest reduction in glucose reduction (51.64%), followed by RW-US (41.95%), whereas the fresh extract showed a considerably lower reduction (11.90%). In contrast, TD-US showed no measurable reduction in glucose levels.

The superior performance of RW drying can be attributed to its infrared radiation-based heat transfer mechanism, which enables rapid and uniform drying at relatively mild temperatures, thereby preserving bioactive compounds associated with hypoglycemic activity. Additionally, compared with conventional methods, its shorter drying time likely minimizes the thermal degradation of key phytochemicals.

The hypoglycemic activity observed in the *B. forficata* extracts in this study is consistent with previous reports on medicinal plants used for diabetes management.

This effect may be related to excessive structural disruption, potentially leading to the release of interfering compounds such as soluble sugars or to partial degradation of bioactive molecules, thereby affecting the measured glucose reduction. Similarly, the lower activity observed in fresh samples may be associated with their high moisture content, which can dilute or hinder the effective interaction between bioactive compounds and the assay system.

The hypoglycemic activity observed in *B. forficata* extracts in this study is consistent with previous reports on medicinal plants used for diabetes management. For example, Andrade and Heinrich [42] reported a 62% glucose reduction for *Opuntia ficus-indica* at 500 µg/mL in this Mexican plant. Da Cunha et al. [43] the antidiabetic potential of *B. forficata* has been linked to flavonoid compounds, particularly kaempferitrin, which has been identified as a key bioactive constituent. These compounds, together with other phenolics, may contribute not only to glucose regulation but also to the mitigation of oxidative stress associated with diabetic conditions.

Da Cunha et al. [43] presented results that show that spray-drying or oven-drying processes applied to *B. forficata* extracts did not significantly alter its flavonoid profile or its hypoglycemic activity. These authors obtained lower values than those of this study because they used different drying processes and conditions.

Overall, the results suggest that RW drying is the most suitable method for preserving hypoglycemic activity in *B. forficata* leaves, highlighting its potential for the development of functional ingredients with antidiabetic properties [44,45,46].

## 3. Materials and Methods

### 3.1. Raw Material

The raw material (*Bauhinia forficata* leaves) was obtained from the central market in Tuxtepec, Oaxaca, Mexico. Leaves were selected and standardized based on their physical attributes. The samples exhibited mean dimensions of 9.0 cm (width), 9.5 cm (length) and a thickness of 0.19 mm, then washed with distilled water and manually sorted to remove foreign matter. The samples were stored at 4 °C prior to processing to minimize degradation. Color was characterized using the hue angle (*h*°), with values ranging from 82 to 84. This selection criterion ensured sample homogeneity in terms of size and color prior to experimental processing. The initial weight of the fresh samples was weighed at the beginning of each drying process, establishing an average weight with three repetitions.

The initial moisture content of the fresh leaves was obtained considering the AOAC oven method [47]. The equilibrium moisture content of the dried samples was obtained by continuously weighing the product over time. Once the weight of the dried sample reached a constant value, the drying time and final moisture content were determined. This final moisture content was considered as the equilibrium moisture content.

### 3.2. Pretreatment of the Raw Material

Ultrasound pretreatment was applied using an ultrasonic processor (Hielscher UP400ST, Teltow, Germany) at a power of 200 W and a frequency of 24 kHz for 1 min prior to the drying treatments [48]. The sonotrode used of the ultrasonicator has the following characteristics: tip diameter of 40 mm, length of 93 mm and material of titanium. The equipment’s power was nominal, it was already supplied by the manufacturer. The applied wavelength was 6.25 cm and the amplitude of the wave peak was 60 μm.

The batch of leaves subjected to ultrasonication consisted of approximately eight leaves per batch, with an average weight of approximately 116 g. These leaves were placed in a 2000 mL beaker containing 1500 mL of water, resulting in a weight-to-volume ratio of 1:15. The *B. forficata* leaves were suspended in the water bath, fully submerged at a controlled temperature of 25 °C, and surrounding the sonotrode. The leaves were individually hung from hooks attached to a metal ring positioned on the surface of the beaker to ensure they did not come into contact with either the sonotrode or the glass surface of the water bath. The ultrasonicated leaves were allowed to dry on absorbent paper for 2 h at room temperature before being used for the various drying treatments.

### 3.3. Drying Kinetics Determination

Two drying systems were employed: a refractance window (RW) dryer and a tray dryer (TD). The RW dryer was constructed at the Food Engineering Laboratory of the National Technological of México, campus Tuxtepec, and its design characteristics have been previously described [27]. The RW system was operated in batch mode using circulating water maintained at 90 °C, resulting in a leaf surface temperature of approximately 70 ± 2 °C. Mylar film with a thickness of 0.036 mm was used as the heat transfer interface between the hot water and the sample.

Tray drying (Polirep, model 3.0, Mexico City, Mexico) was performed under convective conditions at the same product surface temperature at 70 ± 2 °C. Airflow was maintained at a constant velocity of 2 m/s, and relative humidity was not actively controlled but remained within ambient laboratory conditions. Samples were uniformly distributed in a single layer to ensure consistent exposure to the drying medium.

Drying kinetics were monitored gravimetrically at regular time intervals until the equilibrium moisture content (EMC) was reached for each treatment. When the drying line became asymptotic, or parallel to the time line, it was considered to represent the EMC (g water/g dry solids) [10]. The drying kinetics of the samples were obtained by plotting moisture content (g water/g dry solids) versus time (minutes).

All experiments were conducted in triplicate to ensure reproducibility. The treatments evaluated were RW-US (refractance window with ultrasound pretreatment), RW (refractance window), TD-US (tray drying with ultrasound pretreatment), and TD (tray drying).

### 3.4. Physical Properties Determination

#### 3.4.1. Color Measurement of *Bauhinia forficata* Leaves

The dried *B. forficata* leaf samples were reduced in size using a homemade mill (Hamilton Beach, 80393, Richmond, VA, USA) and subsequently sieved through a 50 × 50 mesh screen, obtaining particles of 0.279 mm in size. The fresh *B. forficata* leaf samples were cut into the smallest possible pieces using a 2 mm diameter peeler. To obtain the color parameters, the powdered and fresh samples were placed in mini-Petri dishes 60 mm in diameter by 15 mm high, covering a depth of 14 mm. In both cases, care was taken to ensure that the bottom of the dishes was completely and homogeneously covered with the material up to the test depth.

Color was determined using a colorimeter (3NH, model NR110, Shenzhen, China). The parameters L* (lightness), a* (−green to +red), and b* (−blue to +yellow) in the CIELab scale for all samples. Total color difference (Δ*E*) was calculated employing Equation (1). The instrument was calibrated prior to analysis using standard white and black calibration plates. The equipment used the standard CIE D65 light source (daylight) for the measurements. A 4 mm aperture was used. The illumination geometry was 8/d (diffuse illumination, 8-degree viewing angle). The sensor was positioned vertically above the white or cool light source. This sensor uses an integrating sphere optical path, which eliminates stray light from both the main and auxiliary optical paths. The distance between the measuring aperture and the sample was 2 mm taking into account the height of the unfilled Petri dish and the thickness of the plastic material of aperture’s measuring (1 mm).

The tests were performed three times to obtain repeatability of the results. The units of L*, a*, b* and Δ*E* are dimensionless.

(1) $$∆E=[(L_{s}^{*}-L^{*}{)}^{2}+(a_{s}^{*}-a^{*}{)}^{2}+(b_{s}^{*}+b^{*}{)}^{2}{]}^{1/2}$$

where L* represents lightness (0 = black, 100 = white), a* indicates the chromaticity ranging from green (negative) to red (positive), and b* indicates the chromaticity ranging from blue (negative) to yellow (positive). L_s_*, a_s_*, and b_s_* correspond to the respective CIELab parameters of the fresh *B. forficata* leaves as a standard.

#### 3.4.2. Maximum Shear Force (MSF) and Puncture Force (PF)

Texture analysis was performed using a texture analyzer (Stable Micro Systems, TA-XT Plus, Godalming, Surrey, UK) in cutting and puncture modes. For both tests, the crosshead speed was set at 5.5 mm/s, with a test distance of 40 mm. The MSF and PF were expressed in Newtons (N) and determined as the peak force required to fracture and prick the leaf samples, respectively. The tests were performed three times to obtain repeatability of the results.)

### 3.5. Evaluation of Techno-Functional Properties

#### 3.5.1. Water Absorption Capacity (WAC) and Water Solubility Capacity (WSC)

The WSC is the maximum amount of solute (solid, liquid or gas) that dissolves in water to form a homogeneous mixture, while the WAC is the ability of a material (usually solid) to capture and retain water in its structure, often increasing its weight.

The WAC and WSC were determined according to previously reported methods [49], with slight modifications. Briefly, 1 g of dried sample was mixed with 10 mL of distilled water and vortexed for 30 s. The mixture was then centrifuged (Hettich, Rotina 380R, Tuttlingen, Germany) at 3500× *g* for 15 min. The supernatant was carefully decanted and weighted, then the wet sediment was weighed too; subsequently, this sediment was dried at 105 °C until constant weight, and WAC was calculated using Equation (2) [42].

Subsequently, the collected supernatant divided by the weight of 1 g of dry sample was used to calculate WSC employing Equation (3):

(2) $$\%WAC=\frac{Weight\;of\;wet\;sediment\left(g\right)}{Weight\;of\;dry\;sediment\left(g\right)}\times 100$$

(3) $$\%WSC=\frac{Weight\;of\;supernatant\left(g\right)}{Weight\;of\;dry\;sample\left(g\right)}\times 100$$

#### 3.5.2. Swelling Power (SP)

The SP is the ability of certain materials to absorb water and increase their volume and weight. It is commonly defined as the ratio between the weight of water in the moistened (swollen) sediment and the original dry weight of the material.

A 1 g sample was mixed with 10 mL of distilled water in centrifuge tubes and heated in a water bath at 60, 70, 80, and 90 °C for 30 min. The samples were then centrifuged at 3500 rpm for 15 min. The SP was expressed as the percentage of water retained per gram of dried sample and was calculated using Equation (4):

(4) $$\%SP=\frac{Weight\;of\;wet\;sediment\left(g\right)-Weight\;of\;dried\;sample\left(g\right)}{Weight\;of\;dried\;sample\left(g\right)}\times 100$$

### 3.6. Shrinkage Coefficient Determination

The shrinkage coefficient was determined from geometric measurements of leaf dimensions before and after drying, considering thickness, length, and width. Thickness was measured using a micrometer (Mitutoyo, Series 293, Mexico City, Mexico), and length and width were measured using a digital caliper (Steren, model HER-411, Mexico, City Mexico). The percentage shrinkage for each dimension (thickness, length, and width) was calculated using Equations (5) and (6) based on the initial and final measurements recorded for each sample:

(5) Difference in dimension=Di−Df

(6) $$\%Reduction=\left(\frac{Difference\;in\;dimension}{Di}\right)\times 100$$

where Di is the initial value of the thickness, length or width of the sample before drying (cm), Df is the final value of the thickness, length or width of the sample at the end of drying (cm).

### 3.7. Chemical Properties Evaluation

#### 3.7.1. a_w_ Determination

The water activity (a_w_) levels of fresh and dried *Bauhinia forficata* leaf samples were measured using a temperature-controlled hygrometer (AquaLab, Series 3TE, Pullman, WA, USA) at 25 °C. Prior to analysis, the instrument was calibrated using standard salt solutions according to the manufacturer’s instructions. The samples were allowed to equilibrate in the measurement chamber until a stable reading was obtained. All measurements were performed in triplicate.

It is necessary to establish that a_w_ was measured only at the beginning of the experiment with the moisture content of the fresh samples and at the end of drying with the dry samples of *B. forficata* (considering the final drying time when the equilibrium moisture content is reached) for each treatment used, with or without ultrasound. The units of a_w_ are dimensionless.

#### 3.7.2. Preparation of Extracts

The amount of fresh sample used for extraction was 8.00 g. The leaves were cut into small 2 mm pieces using a peeler. Ultrasound-assisted extraction (UAE) was performed with a solid-to-solvent ratio of 1:10 (*w*/*v*). The extraction was carried out in an ultrasonic bath (Elmasonic P, Singen, Germany) at a frequency of 80 kHz and 100% power for 30 min. A sequential extraction scheme was used with solvents of increasing polarity: hexane, ethyl acetate, methanol, and water, producing four distinct fractions. At each step, the mixture was filtered through Whatman No. 1 filter paper to separate the solid residue from the liquid extract. The solvents were evaporated under reduced pressure using a rotary evaporator (Buchi R-3, Flawil, Switzerland) at 40 °C and 60 rpm. Finally, the extracts were dried to constant weight to determine the extraction yield and stored at 4 °C for later analysis. For the extraction of the dried samples, the amount used was equivalent to that obtained at the drying times when the equilibrium moisture content (EMC) was reached for each drying treatment, which ranged from 1.80 to 1.86 g. The extractions were performed three times for each treatment.

#### 3.7.3. Total Polyphenol Content (TPC)

The TPC was determined using the Folin–Ciocalteu method [44]. A calibration curve was prepared using gallic acid at concentrations of 20, 40, 60, 80, and 100 ppm.

Briefly, 125 µL of sample or standard solution (distilled water as a blank) was mixed with 500 µL of distilled water and 125 µL of Folin–Ciocalteu reagent. The mixture was vortexed and allowed to react for 6 min in the dark. Subsequently, 1.25 mL of 7% (*w*/*v*) Na_2_CO_3_ solution and 1 mL of distilled water were added. The mixture was vortexed again and incubated for 90 min in the dark at room temperature.

The absorbance was measured at 760 nm using a UV–Vis spectrophotometer (Cary 60, Agilent Technologies, Santa Clara, CA, USA). The results were expressed as micrograms of gallic acid equivalents per mL of extract (µg GAE/mL).

#### 3.7.4. Total Flavonoid Content (TFC)

The TFC was determined using the aluminum chloride colorimetric method. Reagents, including 5% (*w*/*v*) NaNO_2_, 10% (*w*/*v*) AlCl_3_, and 1 M NaOH, were prepared and protected from light.

Briefly, 100 µL of extract was diluted with 1000 µL of ethanol. A catechin standard solution (0.1 mg/mL) was used to construct a calibration curve (20–100 µL). To each standard and sample, 1250 µL of distilled water and 75 µL of 5% NaNO_2_ were added and allowed to stand for 6 min. Then, 150 µL of 10% AlCl_3_ was added and incubated for 5 min. Afterward, 500 µL of 1 M NaOH was added, and the volume was adjusted to 2.5 mL with distilled water.

The mixtures were allowed to stand for 30 min at room temperature, and the absorbance was measured at 510 nm using a UV–Vis spectrophotometer (Cary 60, Agilent Technologies, Santa Clara, CA, USA). The results were expressed as catechin equivalents (µg CE/mL).

#### 3.7.5. Antioxidant Activity (DPPH•) Assay

Antioxidant activity was determined using the 2,2-diphenyl-1-picrylhydrazyl (DPPH•) radical-scavenging method [33]. A DPPH• solution was prepared by dissolving 2.4 mg of DPPH• in 100 mL of methanol.

Extracts were prepared at an initial concentration of 10,000 ppm and subsequently diluted to obtain concentrations of 10,000, 5000, 2500, 1000, and 500 ppm. After, 25 µL of sample was mixed with 975 µL of DPPH• solution in light-protected (aluminum-covered) microcentrifuge tubes. The mixtures were incubated for 15 min in the dark at room temperature.

The absorbance was measured at 515 nm using a UV–Vis spectrophotometer (Cary 60, Agilent Technologies, Santa Clara, CA, USA). Antioxidant activity was expressed as a percentage inhibition and calculated using Equation (7) [50]:

(7) $$\%DPPH•Inhibition=\frac{Ca-Sa}{Ca}\times 100$$

where %DPPH• is the Antioxidant activity, Ca is the Control absorbance and Sa is the Sample absorbance.

#### 3.7.6. Antioxidant Activity (ABTS•+) Assay

Antioxidant activity was also evaluated using the ABTS•+ radical cation decolorization assay. The ABTS•+ solution was generated by reacting a 7 mM ABTS•+ stock solution with 2.45 mM potassium persulfate (K_2_S_2_O_8_) and allowing the mixture to stand in the dark at room temperature for 12–16 h. Prior to analysis, the ABTS•+ solution was diluted with ethanol (or phosphate buffer, if applicable) to obtain an absorbance of 0.70 ± 0.02 at 734 nm.

Extracts were prepared at an initial concentration of 10,000 ppm and subsequently diluted to 10,000, 5000, 2500, 1000, and 500 ppm. Briefly, 25 µL of sample was mixed with 975 µL of ABTS•+ solution in light-protected microcentrifuge tubes. The mixtures were incubated for 6 min in the dark at room temperature.

The absorbance was measured at 734 nm using a UV–Vis spectrophotometer (Cary 60, Agilent Technologies, Santa Clara, CA, USA). Antioxidant activity was expressed as a percentage inhibition and calculated using Equation (8) [51]:

(8) $${\%ABTS}^{•+}Inihibition=\frac{Ca-Sa}{Ca}\times 100$$

where %ABTS•+ Inhibition is the Antioxidant activity, Ca is the Control absorbance and Sa is the Sample absorbance.

#### 3.7.7. Chlorophyll Content Determination

Approximately 0.20 g of ground sample was weighed and transferred into aluminum foil-wrapped Falcon tubes to prevent light exposure. Afterward, 2 mL of 95% ethanol was added, and the mixture was vortexed to ensure proper dispersion. The samples were then subjected to extraction in an ultrasonic bath for 35 min, followed by immediate centrifugation (Hettich, Rotina 380R, Tuttlingen, Germany) at 3000 rpm for 10 min at 4 °C.

The supernatant was collected and adjusted to a final volume of 6 mL with 95% ethanol. An aliquot of 1 mL of the extract was further diluted with 7 mL of 95% ethanol. The absorbances of chlorophyll a and chlorophyll b were measured at 665 nm and 649 nm, respectively, using ethanol as the blank. All measurements were performed in triplicate.

The chlorophyll content (µg) was calculated based on the recorded absorbance values using Equations (9)–(11) [52]:

(9) $$Chlorophyll\;a\left(\mu g\right)=13.95\left(A665\right)-6.88\left(A649\right)\times DF\times S$$

(10) $$Chlorophyll\;b\left(\mu g\right)=24.96\left(A649\right)-7.32\left(A665\right)\times DF\times S$$

(11) $$Total\;Chlorophyll\left(\mu g\right)=Chlorophyll\;a+Chlorophyll\;b$$

where A is the absorbance of the chlorophyll extract read at the wavelength indicated by the subscript, DF is the dilution factor and S is the amount of solvent (mL).

### 3.8. Biological Activity Evaluation

#### 3.8.1. Cytotoxicity Assay Determination

The cytotoxicities of the extracts were evaluated using the J774 murine macrophage line. Cells were cultured in RPMI medium supplemented with 10% fetal bovine serum, 100 U/mL penicillin, and 0.1% streptomycin and maintained at 37 °C in a humidified atmosphere containing 5% CO_2_. Cells were seeded at a density of 5 × 10^4^ cells per well in 96-well plates and allowed to adhere for 48 h. Extracts were prepared in dimethyl sulfoxide (DMSO) at a final nontoxic concentration of 0.2%. Subsequently, 20 µL of the test solution was added to each well to achieve the desired concentrations. The plates were incubated for 24 h under standard culture conditions.

After incubation, the culture medium was removed, and 100 µL of serum-free medium (without phenol red) was added to each well. Then, 50 µL of MTT solution [3-(4,5-dimethylthiazol-2-yl)-2,5-diphenyltetrazolium bromide] prepared in calcium- and magnesium-free buffer was added. The plates were incubated for 4 h at 37 °C to allow for formazan crystal formation. Following incubation, the supernatant was carefully removed, and 100 µL of isopropanol was added to dissolve the formazan crystals. The plates were kept at room temperature until complete dissolution. The absorbance was measured at 630 nm using a UV–Vis spectrophotometer (Cary 60, Agilent Technologies, Santa Clara, CA, USA). Cell viability was calculated relative to that of control cells, and the half maximal cytotoxic concentration (CC_50_) was determined by probit analysis [53].

#### 3.8.2. Hypoglycemic Activity Assay

Glucose reduction was quantified using the glucose oxidase–peroxidase (GOD–POD) enzymatic colorimetric method [53]. A glucose stock solution (100 mg/dL) was prepared, and extract solutions were prepared at 10 mg/mL and subsequently diluted to obtain final concentrations of 100, 50, 25, and 12.50 µg/mL.

The assay was performed in a total reaction volume of 1 mL. After, 10 µL of sample was mixed with 1000 µL of glucose oxidase–peroxidase reagent in reaction tubes. The mixtures were incubated at 35 °C for 20 min to allow the enzymatic reaction. The absorbance was measured at 505 nm using a UV–Vis spectrophotometer (Cary 60, Agilent Technologies, Santa Clara, CA, USA). Glucose reduction was determined based on the decrease in absorbance relative to that of the control [54].

### 3.9. Experimental Design and Statistical Analysis

The experiment was conducted using a completely randomized design consisting of four drying treatments: RW-US, RW, TD-US, and TD, each evaluated in triplicate in most of the analyses carried out. Statistical differences among treatments were determined using analysis of variance (ANOVA) followed by LSD mean comparison tests (*p* < 0.05).

## 4. Conclusions

Refractance window drying combined with ultrasound (RW-US) significantly enhanced the drying performance, reduced the processing time and resulted in the lowest water activity (a_w_), indicating improved product stability. The color parameters were well preserved across treatments and remained comparable to those of fresh leaves.

Mechanical analysis revealed that the RW-US and RW treatments resulted in lower maximum shear force values, which were associated with reduced moisture content and increased porosity, whereas higher puncture force (PF) indicated surface densification. Techno-functional properties were maintained or improved, as the water absorption capacity, of the leaf powders was high, whereas the water solubility and swelling power increased in the ultrasound-assisted treatments.

Shrinkage was predominantly observed in thickness, reflecting structural collapse during drying. RW-US also promoted higher retention and extractability of polyphenols and flavonoids, which was consistent with the enhanced antioxidant activity observed in both the DPPH• and ABTS•+ assays. Additionally, the chlorophyll content was better preserved under ultrasound-assisted conditions.

Importantly, none of the extracts exhibited cytotoxic effects on J774 cells, confirming their safety. The hypoglycemic activity levels were highest in the RW, RW-US, and TD treatments, highlighting the potential of these processes to preserve bioactive compounds with antidiabetic properties.

Overall, RW-US emerges as the most effective drying strategy, offering a favorable balance between process efficiency, structural preservation, and functional and biological properties, supporting its application in the development of functional food ingredients from *Bauhinia forficata* leaves.

## Figures and Tables

**Table 1:** Effect of drying treatments on color parameters, water activity, and equilibrium moisture content of *Bauhinia forficata* leaves.

Treatment	L*	a*	b*	∆*E*	a_w_	EMC (g Water/g Dry Solids)	Time (min)
Fresh	12.77 ± 0.81 ^a^	4.26 ± 0.71 ^ab^	35.13 ± 0.65 ^ab^	-	0.92 ± 0.0 ^a^	-	-
RW-US Drying	13.04 ± 0.14 ^a^	3.93 ± −0.18 ^a^	35.57 ± 0.09 ^a^	0.61 ± 0.1 ^a^	0.21 ± 0.01 ^b^	0.2915 ± 0.03 ^a^	90 ± 2.35 ^a^
RW Drying	12.90 ± 0.04 ^a^	4.67 ± 0.06 ^b^	34.84 ± 0.08 ^b^	0.52 ± 0.1 ^a^	0.28 ± 0.02 ^c^	0.3086 ± 0.02 ^a^	120 ± 3.89 ^b^
TD-US Drying	11.39 ± 0.45 ^b^	5.72 ± 0.54 ^c^	35.14 ± 0.43 ^ab^	2.01 ± 0.28 ^b^	0.29 ± 0.01 ^c^	0.3084 ± 0.01 ^a^	120 ± 4.92 ^b^
TD Drying	8.00 ± 0.03 ^c^	9.94 ± 0.05 ^d^	30.05 ± 0.04 ^c^	8.99 ± 0.30 ^c^	0.36 ± 0.01 ^d^	0.3098 ± 0.02 ^a^	180 ± 5.10 ^c^

Values are mean ± standard deviation (n = 3). Different lowercase letters in the same column indicate significant differences (LSD, *p* < 0.05). The units of L*, a*, b*, ∆*E* and a_w_ are dimensionless.

**Table 2:** Absorption capacity, water solubility and swelling power in *B. forficata* leaves.

	Swelling Power (%)					
Treatment	60 °C	70 °C	80 °C	90 °C	WAC (g agua/g d.m.)	WSC (%)
RW-US Drying	9.02 ± 0.63 ^aA^	9.38 ± 0.60 ^aA^	9.76 ± 0.23 ^aA^	9.95 ± 0.63 ^aA^	5.56 ± 0.12 ^a^	13.75 ± 0.02 ^a^
RW Drying	8.51 ± 0.48 ^aA^	9.21 ± 0.18 ^aA^	9.39 ± 0.46 ^aA^	9.61 ± 0.47 ^aA^	5.35 ± 0.10 ^a^	11.08 ± 0.01 ^b^
TD-US Drying	7.80 ± 0.61 ^bA^	8.13 ± 0.09 ^bAB^	9.13 ± 0.52 ^aC^	9.27 ± 0.30 ^aC^	5.55 ± 0.06 ^a^	12.11 ± 0.01 ^b^
TD Drying	6.77 ± 0.31 ^bA^	7.46 ± 0.43 ^bA^	8.46 ± 0.43 ^bA^	8.85 ± 0.52 ^bB^	5.36 ± 0.10 ^a^	9.76 ± 0.01 ^c^

d.m. = dry matter. Values are the mean ± standard deviation (n = 3). Different lowercase letters in the same column indicate significant differences (LSD, *p* < 0.05). Different uppercase letters in the same row indicate significant differences (LSD, *p* < 0.05).

**Table 3:** Effect of drying treatments on the shrinkage coefficient of *Bauhinia forficata* Leaves.

Treatment	Thickness Reduction (%)	Width Reduction (%)	Length Reduction (%)
RW-US Drying	32.42 ± 0.08 ^a^	3.79 ± 0.02 ^a^	4.73 ± 0.01 ^a^
RW Drying	30.65 ± 0.09 ^b^	3.35 ± 0.03 ^b^	4.05 ± 0.04 ^b^
TD-US Drying	32.12 ± 0.06 ^c^	3.84 ± 0.01 ^c^	4.70 ± 0.01 ^c^
TD Drying	31.28 ± 0.07 ^d^	3.55 ± 0.04 ^d^	4.23 ± 0.02 ^d^

Values are expressed as mean ± standard deviation (n = 3). Different lowercase letters within the same column indicate statistically significant differences (LSD test, *p* < 0.05).

**Table 4:** Effect of different drying treatments on the content of polyphenols, flavonoids and chlorophyll in the leaves of *B. forficata*.

Treatment	Total Polyphenol Content mg GAE/g Sample *	Total Flavonoid Content mg QE/g Sample *	Chlorophyll (μg/g Sample) *	Retention Chlorophyll (%)
Fresh	43.79 ± 0.77 ^a^	126.62 ± 1.50 ^a^	1.59 ± 0.04 ^a^	---
RW-US Drying	61.96 ± 0.61 ^b^	308.44 ± 5.02 ^b^	2.65 ± 0.01 ^b^	66.66 ± 3.10 ^a^
RW Drying	33.67 ± 0.87 ^c^	260.93 ± 1.15 ^c^	1.88 ± 0.02 ^c^	18.23 ± 1.30 ^b^
TD-US Drying	37.34 ± 1.11 ^d^	280.40 ± 1.32 ^d^	2.03 ± 0.04 ^d^	27.67 ± 1.30 ^c^
TD Drying	5.26 ± 0.23 ^e^	82.80 ± 0.88 ^e^	1.72 ± 0.01 ^e^	08.61± 1.20 ^d^

Values are mean ± standard deviation (n = 3). Different lowercase letters in the same column indicate significant differences (LSD, *p* < 0.05). * Chlorophyll content, total phenolic content (TPC), and total flavonoid content (TFC) in fresh leaves were determined as μg/g sample mass (wet basis), whereas in dried leaves they were determined as μg/g dry mass (dry basis).

**Table 5:** Cytotoxicity and hypoglycemic activity values of *Bauhinia forficata* leaves under the different drying treatments.

Treatment	Cytotoxicity CC_50_ (µg/mL)	Hypoglycemic Activity Concentration (mg/dL)	Glucose Reduction (%)
Fresh	>200	176.20 ± 5.02	11.90
RW-US Drying	>200	116.10 ± 2.28	41.95
RW Drying	>200	96.72 ± 3.14	51.64
TD-US Drying	>200	200.00	0.00
TD Drying	>200	120.59 ± 4.16	39.70

Control = 200 mg/dL. Extract concentration 100 μg/mL. Values are the mean ± standard deviation (n = 3).

## Data Availability

The original contributions presented in this study are included in the article. Further inquiries can be directed to the corresponding author(s).
